# Compensatory Mechanisms of Pancreatic Beta Cells: Insights into the Therapeutic Perspectives for Diabetes 

**DOI:** 10.1155/2014/217387

**Published:** 2014-07-17

**Authors:** Romano Regazzi, Stephane Dalle, Amar Abderrahmani

**Affiliations:** ^1^Department of Fundamental Neurosciences, Faculty of Biology and Medicine, University of Lausanne, Rue du Bugnon 9, 1005 Lausanne, Switzerland; ^2^Institute of Functional Genomic, INSERM 661, CNRS 5203, Montpellier 1 and 2 Universities, Montpellier, France; ^3^Lille 2 University and European Genomic Institute for Diabetes (EGID) FR 3508, University of Lille Nord de France, CNRS-UMR 8199, Lille, France

Diabetes is one of the leading causes of premature mortality worldwide and has become one of the major health challenges of the 21st century. Diabetes develops when insulin production is insufficient to compensate for the increased insulin requirements elicited by insulin resistance. Diabetes prevalence has risen dramatically during the last 20 years in parallel to the pandemic of obesity. Although obesity is the first diabetes risk factor, many obese and insulin resistant people do not suffer from diabetes. This situation is thought to result from the capacity of beta cell to adapt its mass and function in order to produce enough insulin to cover the needs of the organism. Beta cell adaptation is a physiological process that occurs efficiently from birth to early childhood periods and during pregnancy. Beta cell functional mass adaptation relies on both increased intrinsic insulin secretory capacity of the cell and beta cell mass expansion and preservation of cell survival against apoptosis. Deciphering the key mechanisms that account for such beta cell plasticity would help understanding the pathophysiology of diabetes, paving the way for the generation of innovative antidiabetic compounds aiming at preventing the development and the progression of the disease. In this respect, this special issue contains two regular articles and four reviews that highlight some of the mechanisms involved in the control of beta cell mass and function.

Beta cell mass adaptation relies on neogenesis and, possibly, on an increase in beta cell proliferation. Although so far only few studies have confirmed a consistent replication of human adult beta cells, this phenomenon has been demonstrated to play a major role in rodent beta cell mass expansion. Beta cell proliferation involves a tight control and interconnectedness in the expression of activators and inhibitors of the cell cycle. In this special issue, the paper entitled “*Role of Ink4a/Arf locus in beta cell mass expansion under physiological and pathological conditions*” by E. Salas et al. reports the pieces of evidence pointing to a role for the two tumor suppressors p16^ink4a^ and p14^Arf^ encoded by the Ink4a/Arf in beta cell expansion. In this paper, the authors provide an insightful description of their mode of action and their regulation under diabetogenic and physiological conditions. Induction of the unfolded protein response, also referred to as endoplasmic reticulum stress (ER), is a key cellular phenomenon that governs beta cell compensation. Induction of ER stress is required for the effects of glucagon like-peptide 1 (GLP-1) on the potentiation of glucose-induced insulin secretion and beta cell survival. The review entitled “*Role of the unfolded protein response in *β* cell compensation and failure during diabetes*” by N. Rabhi et al. sheds light on the key signaling pathways of the ER stress involved in the regulation of beta cell function and survival under physiological and diabetes condition.

Mechanisms involved in the control of glucose-induced insulin secretion are attractive targets for antidiabetic therapy. Closure of ATP-dependent potassium channel is a key event that is required for glucose-induced insulin secretion. This channel is closed by glinides, a class of oral antidiabetic agents. As a consequence of the drug action, the sensitivity of beta cells to elevated glucose levels is increased and, thereby, the early-phase prandial insulin response is enhanced. The goal of the paper entitled “*Comparison of metformin and repaglinide monotherapy in the treatment of new onset type 2 diabetes mellitus in China*” by J. Ma et al. is to compare the efficiency of metformin and repaglinide monotherapies in the glycemic control of newly diagnosed diabetic Chinese patients. The study reports a decrease of glycated hemoglobin and, finally, a better glycemic control in the diabetic patients treated with repaglinide when compared to metformin. This beneficial effect is associated with increased beta cell function. This paper underlines the effectiveness of approaches targeting the beta cell as the first medication to rapidly improve the glycemic control. While the classes of stabilized GLP-1 mimetics and dipeptidyl peptidase-IV inhibitors aim to potentiate glucose-induced insulin secretion by triggering the GLP-1 receptor signaling, they are not first-line drugs for lowering blood glucose. However, there are numerous clues underlining their potent effects in preventing a steady decline of beta cell functions overtime and, consequently, the progression of diabetes. Increased GLP-1 levels may contribute to functional beta cell mass adaptation in obesity and pregnancy and, importantly, may also protect beta cells against the harmful effects of diabetogenic factors including proinflammatory cytokines. The beneficial effects of incretins such as GLP-1 on beta cells are thought to represent also one of the important mechanisms whereby bariatric surgery in obese diabetic patients permits to improve glycemia aside from massive weight loss. The H. Ezanno et al. paper entitled “*JNK3 is required for the cytoprotective effect of exendin 4*” highlights the key role of the beta cell restrictive c-Jun amino terminal kinase 3 (JNK3) in relaying the protective effect of the exendin 4 GLP-1 mimetic against cell death elicited by proinflammatory cytokines. The study provides some lines of evidence that JNK3 could be an attractive drug target.

Induction of the inducible cAMP early repressor (ICER) is part of the adaptive mechanisms through which GLP-1 and its mimetics allow insulin secretion to return to basal state. ICER is a passive repressor that inhibits the expression of genes containing a cAMP response element (CRE) in their promoter. Upon silencing its target genes, the level of ICER needs to be reduced for preserving beta cell integrity. Several studies reviewed in the paper entitled “*Decompensation of *β*-cells: when pancreatic *β*-cells are on ICE(R)*” by R. Salvi and A. Abderrahmani have shown that abnormal expression of ICER underlies dysfunction and death of insulin-secreting cells under diabetogenic conditions, thus emphasizing the requirement for a tightly controlled expression of ICER for beta cell compensation.

The literature demonstrating a role for microRNAs in beta cell compensation during obesity and pregnancy is emerging. In this special issue, the paper entitled “*Role of microRNAs in islet beta-cell compensation and failure during diabetes*” by V. Plaisance et al. provides an overview of the potential involvement of these small noncoding RNAs in beta cell mass expansion and failure occurring during normal development and in response to metabolic changes and diabetogenic conditions including the exposure to cytokines, oxidized LDL, and palmitate.

*Romano Regazzi* *Romano Regazzi* *Stephane Dalle* *Stephane Dalle* *Amar Abderrahmani* *Amar Abderrahmani*

